# A Pseudorange Measurement Scheme Based on Snapshot for Base Station Positioning Receivers

**DOI:** 10.3390/s17122783

**Published:** 2017-12-01

**Authors:** Jun Mo, Zhongliang Deng, Buyun Jia, Xinmei Bian

**Affiliations:** School of Electronic Engineering, Beijing University of Posts and Telecommunications, No. 10 Xitucheng Road, Haidian District, Beijing 100876, China; dengzhl@bupt.edu.cn (Z.D.); jiabuyun@bupt.edu.cn (B.J.); bianxinmei@bupt.edu.cn (X.B.)

**Keywords:** CMMB, pseudo code, TDOA, Taylor expansion

## Abstract

Digital multimedia broadcasting signal is promised to be a wireless positioning signal. This paper mainly studies a multimedia broadcasting technology, named China mobile multimedia broadcasting (CMMB), in the context of positioning. Theoretical and practical analysis on the CMMB signal suggests that the existing CMMB signal does not have the meter positioning capability. So, the CMMB system has been modified to achieve meter positioning capability by multiplexing the CMMB signal and pseudo codes in the same frequency band. The time difference of arrival (TDOA) estimation method is used in base station positioning receivers. Due to the influence of a complex fading channel and the limited bandwidth of receivers, the regular tracking method based on pseudo code ranging is difficult to provide continuous and accurate TDOA estimations. A pseudorange measurement scheme based on snapshot is proposed to solve the problem. This algorithm extracts the TDOA estimation from the stored signal fragments, and utilizes the Taylor expansion of the autocorrelation function to improve the TDOA estimation accuracy. Monte Carlo simulations and real data tests show that the proposed algorithm can significantly reduce the TDOA estimation error for base station positioning receivers, and then the modified CMMB system achieves meter positioning accuracy.

## 1. Introduction

As the main positioning technology, global navigation satellite systems (GNSS) have many advantages, such as large coverage area, high positioning accuracy, and fine robust [1]. However, due to the loss of GNSS signals by obstructions from buildings, the positioning effectiveness and accuracy of the GNSS are limited in urban canyons and indoor environments [2,3]. Therefore, terrestrial radio positioning systems and their enhancements to the GNSS are got increasing attention. 

**Figure 1:**
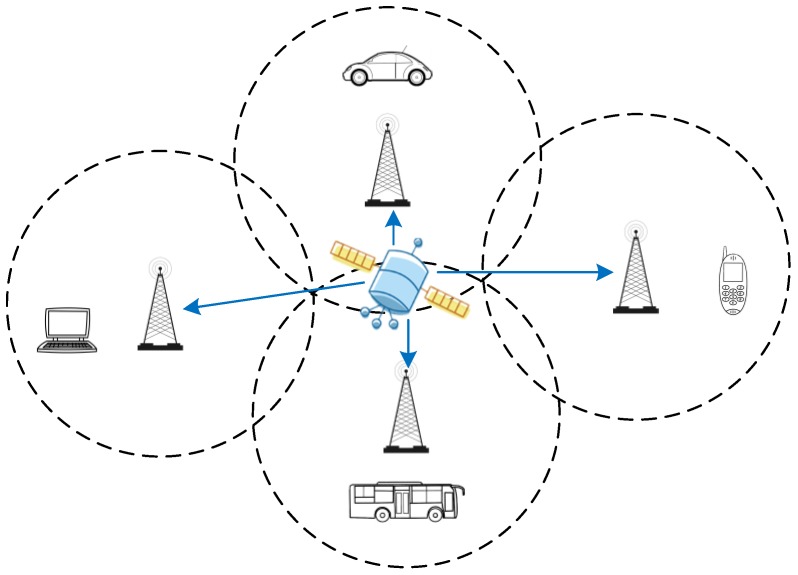
Terrestrial single frequency network coverage of the China mobile multimedia broadcasting (CMMB) system.

Recently, with the rapid development of data and multimedia services, digital multimedia broadcasting technologies have been widely used. This paper mainly studies a digital multimedia broadcasting technology, named China mobile multimedia broadcasting (CMMB). It has been recognized that novel wireless positioning methods can be designed by utilizing the CMMB signal [4,5]. When compared with the GNSS, the positioning technology based on CMMB signals has many potential advantages: the signal transmission power is stronger and the frequency band is U Band (470–798 MHz) [6], which contributes to better urban or indoor propagation than the GNSS of L Band. However, the CMMB standard did not take account the use of CMMB signals for location based service (LBS) at the beginning of the design. The CMMB system mainly adopts single frequency network (SFN) to achieve ground network coverage, and the receiving sensitivity of CMMB receivers is −95 dBm [6]. Figure 1 shows the terrestrial SFN coverage of the CMMB system, and the base station receives and forwards the multimedia data transmitted by the broadcast satellite. The receiver must receive three or more CMMB base station signals in order to achieve high accuracy positioning, but with the existing CMMB receivers it is difficult to receive more than three CMMB signals in the SFN model. In addition, the CMMB system cannot provide the basic positioning information of the base station, such as base station location, height, and delay correction [7]. Based on these facts, it is difficult to achieve high accuracy indoor and outdoor seamless positioning by using the existing CMMB signal.

**Figure 2:**
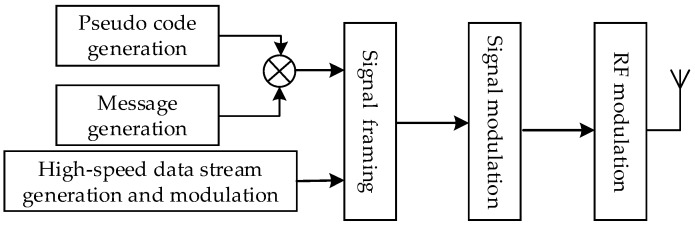
Flowchart of the signal generation.

In order to achieve high accuracy indoor and outdoor positioning, the existing CMMB system should be modified. Pseudo codes are used as the ranging signal in the pseudorange measurement, whose spread spectrum gain is higher than that of the multimedia broadcasting signal. Then, pseudo codes have larger effective coverage area than the multimedia broadcasting signal. Hence, the modified CMMB system is multiplexing the communication signal and pseudo codes in the same frequency band, and pseudo codes are used for positioning. When transmitting the fusion signal, we control the transmit power of PRN codes to prevent the impact of PRN codes on the communication of CMMB signals. When the power of the CMMB signal is higher than that of PRN codes by 18 dB or more, then the CMMB receiver can ignore the impact of PRN codes and perform normal demodulation. The existing deployment of the CMMB system does not change, only some other equipment are added on the original basis, and the positioning service can be done. The positioning part of the modified CMMB system is a direct-sequence spread spectrum code division multiple access (DSSS-CDMA) system, employing binary phase shift keying (BPSK) modulation. The basic information of the base stations for positioning is also added, which consists of coordinated universal time (UTC), base station number, air pressure, and base station coordinates. Figure 2 shows the flowchart of the signal generation of the modified system. The signal bandwidth of base station receivers is designed 8 MHz, which is much larger than the chipping rate of GNSS signals. So, the accuracy of the pseudo code range measurements can be improved. In contrast to the GNSS, the range between the base stations and the receivers changes very slowly in the indoor environment. So, the positioning signal cannot experience significantly the Doppler effects and the impairment that is caused by the delay of ionosphere propagation, which will lead to easier signal acquisition [8]. Therefore, high accuracy positioning can be achieved by adding some simple modifications to the deployed CMMB facilities.

In the modified CMMB system, the time difference of arrival (TDOA) is the main pseudorange measurement method and the key to achieve high accuracy positioning. Due to pseudorange codes being added in the modified CMMB system, the signals from different stations can be tracked using similar GNSS receiver tracking algorithms. When signal-to-noise ratio (SNR) is ideal and the propagate channel is stable, the accurate code phase difference is obtained by tracking algorithms, and then TDOAs are obtained. However, in the complex fading channel and low SNR, the signal strength violently fluctuates, and the traditional tracking loop is difficult to maintain the stability of the signal tracking. Then, the tracking channel cannot accurately output the pseudo code phase of the base station signal to the receiver, and the TDOA cannot be retrieved. Especially for signals from the far base station, the mean time to lose lock (MTTL) rises and continuing tracking is difficult [9]. There are many studies to solve the complex fading channel problems [10,11,12,13]. In Reference [10], the algorithm of the radio propagation path under non-line-of-sight (NLOS) is studied to improve the positioning accuracy of the time of arrival (TOA), but the process of obtaining TOA is not described in detail. The rake receiver that is described in [11] can improve the time of stable tracking in the fading channel, but it has not studied how to use it for high accuracy ranging. Refs. [12,13] proposed the signal tracking loop architecture and the loop adjustment algorithm in the fading channel, which can reduce the average lockout time of the loop under the Rayleigh channel. But, the premise of the above algorithms is accurate channel estimation, otherwise the accurate TOA cannot be an output. In addition, the limited bandwidth of receivers also affects the accuracy of the TDOA estimation, thus affecting the positioning accuracy. Refs. [14,15] improve the code phase estimation accuracy of band-limited receivers using curve fitting, but these algorithms require more observations and the fitting accuracy is limited. The non-causal smoothing estimator is added to improve the measurement accuracy in snapshot GNSS receivers [16,17], but the premise of the algorithm is that the weak signal must be stable tracking. This paper proposes a novel pseudorange measurement algorithm for positioning in the fading channel. The basic idea of the proposed algorithm is to extract the TDOA estimation from the stored signal fragments, and to utilize the Taylor expansion of the autocorrelation function to get the accuracy TDOA. The receiver takes a snapshot of the receiving signal at the appropriate time, and then analyzes the stored signal by replay to get the TDOAs.

The rest of this paper is organized as follows. In Section 2, the fusion signal model, including the CMMB signal and pseudo codes, is given and the autocorrelation function with band-limited is analyzed. Section 3 presents a pseudorange measurement scheme that is based on snapshot and the Taylor expansion of the autocorrelation function. Section 4 gives simulation results, real data tests and performance analysis of the proposed algorithm. Finally, the conclusions are shown in Section 5.

## 2. Fusion Signal Model and Autocorrelation Function with Band-Limited

In this section, a novel fusion signal is designed that multiplexes communication signal and pseudo codes in the same frequency band. The autocorrelation function with band-limited is described and analyzed. 

### 2.1. Fusion Signal Mode

**Figure 3:**
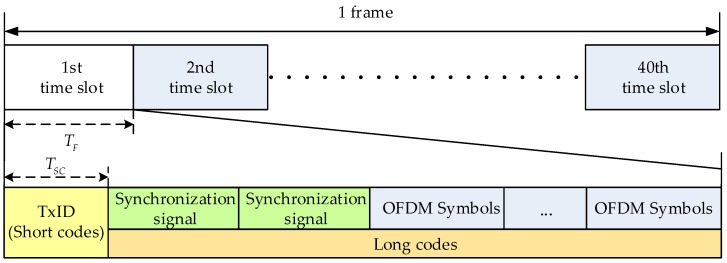
Structure of the fusion signal.

The CMMB system transmits the digital multimedia broadcasting signal using orthogonal frequency division multiplexing (OFDM) technology [18]. One frame of the CMMB signal is one second, and is divided into 40 time slots. Each time slot contains one beacon and 53 OFDM symbols. At the beginning of the CMMB signal design, the beacon includes a transmitter identification signal named TxID, and two identical synchronous signals. The TxID signal is the number of the corresponding base station, and the synchronous signals and the transmitted data symbols are the same. However, the TxID signal in the existing CMMB signal is empty, and the base station receivers are not concerned with the TxID. Even if the base station receivers receive a large number of the CMMB signals at some point, it is impossible to distinguish the different signals from different base stations. Therefore, pseudo codes and the CMMB signal are multiplexed in the same frequency band, and then the modified CMMB system can provide high accuracy positioning services for us. Pseudo codes are divided into two kinds to be superimposed on the CMMB signal, the longer pseudo codes are called long codes and the short ones are called short codes, as shown in Figure 3. Due to the TxID being empty, short codes and the CMMB signal have the same transmit power. In order not to affect the normal communication of the CMMB signal, the power of long codes is lower than the CMMB signal 20 dB. The length of one bit positioning data is the same as the length of one time slot broadcasting signal, and the time slot of pseudo codes is perfectly aligned with the CMMB signal. The CMMB system and the positioning system can share the same 1 pps pulse signal to adjust the fusion signal broadcasting, and then it achieves the synchronization between the modified base stations. Because the power of short codes is stronger than that of long codes, the receiver is acquired with short codes and is tracked with short and long codes.

The novel fusion signal of the *k*th time slot is expressed as:

(1) $$s_{k}^{\left(i\right)}(t)=\;\left\{ \begin{array}{ll} s_{CMMB}(t)+c_{SC}^{\left(i\right)}(t) & (k-1)T_{F}\leq t<(k-1)T_{F}+T_{SC}\\ s_{CMMB}(t)+\alpha c_{LC}^{\left(i\right)}(t) & (k-1)T_{F}+T_{SC}\leq t\leq kT_{F}\\ 0 & others \end{array}\right.$$

where, superscript *i* stands for the base station number, $s_{CMMB}(t)$ is the CMMB signal, $c_{SC}(t)$, and $c_{LC}(t)$ denote short codes and long codes, respectively, $T_{F}$ is the time length of the time slot, $T_{SC}$ is the time length of the short codes. 

The novel signal of *i*th base station is:

(2) $$s^{\left(i\right)}(t)=\;\sum_{k=-\infty }^{\infty }d_{k}^{\left(i\right)}(t)s_{k}^{\left(i\right)}(t)$$

where *d_k_*(*t*) denotes the positioning data. The transmitting signal of *i*th base station is:

(3) $$S^{\left(i\right)}(t)=\;s^{\left(i\right)}(t)\cos(2\pi f_{c}t+\varphi_{0,i}(t))$$

where $f_{c}$ is the carrier frequency, $\varphi_{0,i}(\cdot )$ is the initial phase.

Thanks to the orthogonality of pseudo codes, the received signals from base stations can be analyzed separately [19]. The fusion signal is received by radio frequency (RF) antenna of the receivers, and the output of RF antenna is expressed as:

(4) $$r(t)=\;\sum_{i=1}^{N}A^{\left(i\right)}s^{\left(i\right)}(t-\tau_{i})\cos(2\pi (f_{c}+f_{d,i})t+\varphi_{0,i}(t))+\omega (t)$$

where *N* is the number of the received signals from *N* different base stations, $A^{\left(i\right)}$ is the signal amplitude, $\tau_{i}$ is the incoming signal delay, $f_{d,i}$ is the incoming Doppler shift, ω(t) stands for the additive Gaussian white noise (AWGN) component with zero mean (μ=0) and variance ($\sigma_{n}^{2}$). The output signal of RF antenna is converted to intermediate frequency (IF) through the amplifier, mixer, and filter, and finally IF signal is output to the baseband processor by the analog-to-digital converter (ADC) model. The CMMB signal is filtered out. Neglecting the quantization effect, the incoming signal of the baseband processor can be expressed as:

(5) $$r(nT_{s})=\sum_{i=1}^{N}A_{ADC}^{\left(i\right)}c_{P}^{\left(i\right)}(nT_{s}-\tau_{i})e^{j2\pi (f_{IF}+f_{d,i})nT_{s}+\varphi_{0,i}}+\omega (n)$$

where $A_{ADC}^{\left(i\right)}$ is the signal amplitude after ADC, $T_{s}$ is sampling time, $f_{IF}$ denotes the intermediate frequency, $c_{P}(\cdot )$ is the positioning signal, and $r(n)=r(nT_{s})$.

### 2.2. Autocorrelation Function with Band-Limited

**Figure 4:**
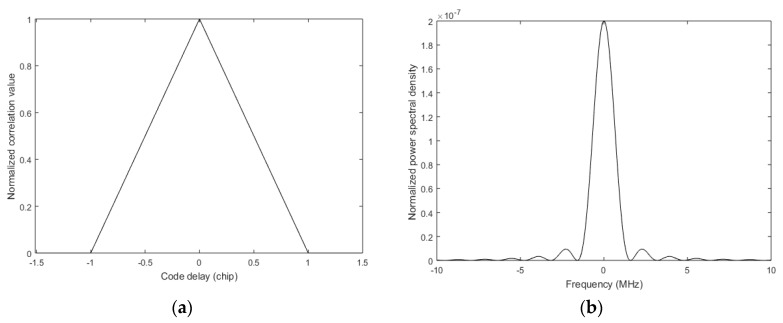
Characteristics of the autocorrelation function: (**a**) is autocorrelation function with infinite bandwidth; and, (**b**) is power spectral density.

We can only use the autocorrelation function to distinguish the signals from different modified base stations. When pseudo codes have these two characteristics of the infinite period and the ideal autocorrelation, the autocorrelation function of $c_{P}(t)$ can be expressed as:

(6) $$R_{P}(\tau )=\{\begin{array}{cc} 1-\left|\tau \right|/T_{c} & \left|\tau \right|\leq T_{c}\\ 0 & others \end{array}$$

where τ is the incoming signal delay, $T_{c}$ is the chip period, and the shape of the correlation peak is shown in Figure 4a. The normalized power spectral density of $c_{P}(t)$ is

(7) $$G_{P}(f)=T_{c}\sin c^{2}(\pi fT_{c})$$

and the shape of $G_{P}(f)$ is shown in Figure 4b.

In the modified CMMB system, the positioning signal is filtered into the same bandwidth as the CMMB signal. At the same time, the bandwidth of the base station receiver is limited. On the one hand, filtering leads to a loss of signal power; on the other hand, the shape of the autocorrelation function in the receiver is not the triangle given by Equation (6). The frequency response of the ideal low pass filter is

(8) $$H_{L}(f)=\{\begin{array}{cc} 1 & \left|f\right|\leq B/2\\ 0 & others \end{array}$$

where *B* is the RF bandwidth of the receiver. The proportion of the signal power that can pass through the part of the filter is

(9) $$\eta =\int_{-B/2}^{B/2}G_{P}(f)df$$

**Figure 5:**
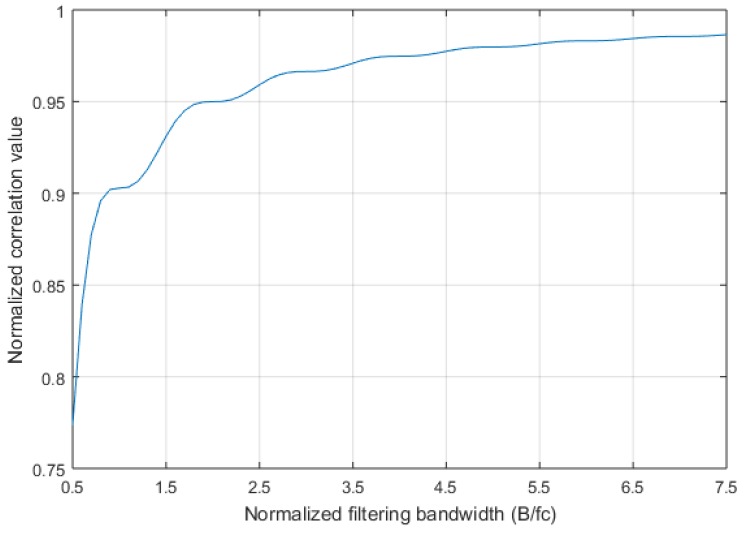
The proportion of the signal component through the filter in the total signal under different bandwidths.

Figure 5 illustrates the proportion of the signal component through the filter in the total signal under different bandwidths, and chip rate $f_{c}=1/T_{c}$.

In the receiver, acquisition and tracking are based on the autocorrelation between the incoming signal and the local replica codes [20,21]. Assuming that the incoming signal is exactly the same modulation scheme and spread spectrum sequences as the local replica codes, the differences are that the incoming signal passes through the filter defined by Equation (8) and the local replica codes are not filtered. Then, the autocorrelation function is

(10) $$R_{L}(\tau )=\int_{-B/2}^{B/2}G_{P}(f)e^{j2\pi f\tau }df$$

**Figure 6:**
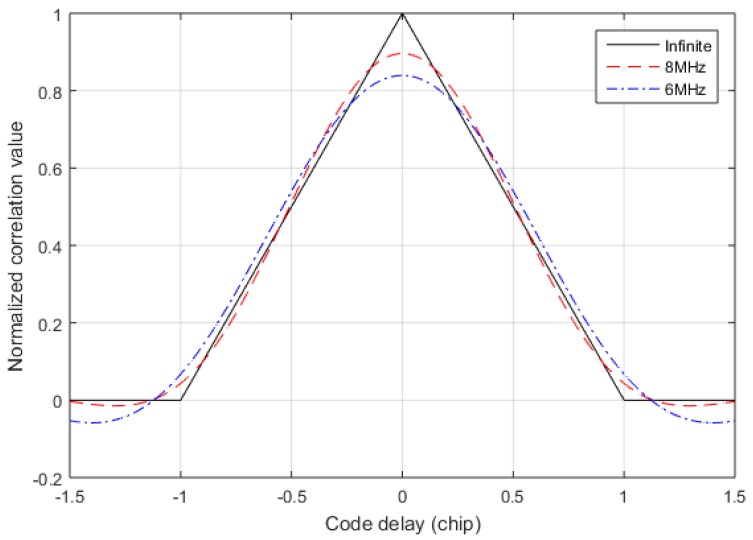
The autocorrelation function under different bandwidths.

$R_{L}(\tau )$ under several different filtering bandwidths is shown in Figure 6, and $f_{c}=5\;\mathrm{MHz}$. It can be found that when the filtering bandwidth is narrow, the autocorrelation function becomes smooth near τ=0.

## 3. A Pseudorange Measurement Scheme Based on Snapshot

Since continuous positioning signals exist, it makes the receiver possible to track the positioning signals from different base stations with regular GNSS-like algorithms, and the receiver contains several tracking channels to get TDOAs. However, in the fading channel, due to the violent fluctuations of the signal strength, the regular tracking loop is difficult to maintain the stability of the signal tracking, or even the losses of lock. Then, the tracking channel cannot output the accurate pseudo-code phase, and TDOAs cannot be obtained. In this section, a pseudorange measurement scheme that is based on snapshot is proposed to solve the above problem. When considering the impairment caused by the limited bandwidth, Taylor expansion is utilized to improve the pseudorange accuracy.

### 3.1. Pseudorange Measurement Scheme

The basic idea of this scheme is to abandon the pseudorange measurement method in the traditional tracking mode, and to use the stored signal fragment to extract TDOAs. The premise of the algorithm is that the receiver has stably tracked a strong positioning signal. The receiver takes a snapshot of the received signal at the appropriate time, and analyzes the stored data by replay to obtain TDOAs. As long as the snapshot data is not impaired by the fading channel, then the receiver can output the right pseudorange.

**Figure 7:**
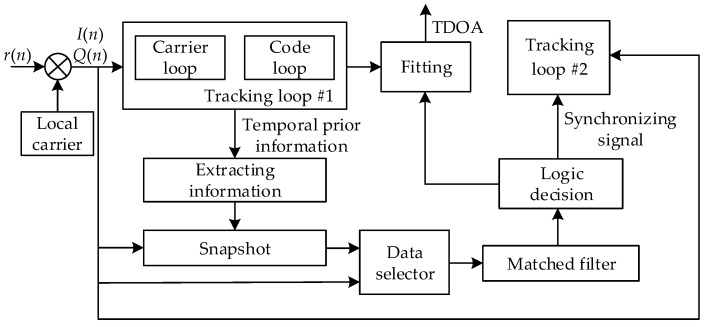
Flowchart of the proposed algorithm.

**Figure 8:**
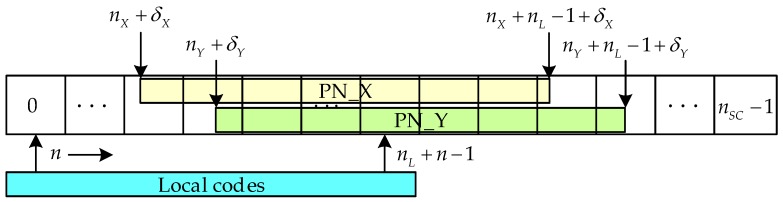
Flowchart of the proposed algorithm.

The whole procedure of the proposed algorithm is shown in Figure 7. When the receiver starts, the data selector selects the path form the in-phase (I) or quadrature (Q) signals to the matched filter. The matched filter searches for the short codes of all the possible base station signals. The logic decision module performs an acquisition decision on the signal from the nearby base station and outputs the synchronize information for the late successful tracking. From the periodicity of the positioning signal, as shown in Equation (1) and Figure 3, when a strong positioning signal is stably tracked, the temporal prior information can be extracted to determine the time of arrival of the next short codes. At the moment of the arrival of the next short codes, I/Q data of short codes is stored in the memory and the storage time is $T_{SC}$, and the snapshot is completed. If the sampling rate of the receiver is $f_{s}$, then the length of the stored I/Q data is

(11) $$n_{SC}=\left\lceil T_{SC}f_{s}\right\rceil$$

and $n_{sc}$ is longer than the length of the matched filter $n_{L}$. After the snapshot, the stored data is correlated with the local codes in the matched filter, and the output of the matched filter can be expressed as

(12) $$V(n)=\sqrt{{\left(\sum_{k=n}^{n+n_{L}-1}I(k)c_{L}(k)\right)}^{2}+{\left(\sum_{k=n}^{n+n_{L}-1}Q(k)c_{L}(k)\right)}^{2}}=A_{V}R_{L}(\tau )+w(n)$$

where I(k) and Q(k) are in-phase and quadrature signals, respectively, $c_{L}(k)$ is the local code, is the amplitude of the stored signal, w(⋅) is the noise distributed by Rayleigh. For the convenient of discussion, it is assumed that the receiver receives the PN sequence *X* and *Y* of the short codes. Since there is a difference in the arrival time between *X* and *Y*, their locations in the memory are in order, as shown in Figure 8. Each memory address represents the relative location of each sample point in the discrete time domain.

The logic decision is used to find the correlation peak and transmit the correlation results near the correlation peak to the correlation peak fitting module. The fitting module fits the location of the correlation peak to obtain the relative relationship between the correlation peak and the memory address. In Figure 8, the start location of the PN sequence X is located between the memory address $n_{X}$ and $n_{X}+1$, with the offset $\delta_{X}$. The following equations can be listed according to Equation (12).

(13) $$\{\begin{array}{c} V(n_{X})=A_{V}R_{L}(\delta_{X})\\ V(n_{X}+1)=A_{V}R_{L}(1/f_{s}-\delta_{X}) \end{array}$$

If $R_{p}(\cdot )$ is used instead of $R_{L}(\cdot )$, $\delta_{X}$ can be expressed as

(14) $$\delta_{X}=\frac{V(n_{X}+1)f_{s}+V(n_{X})(f_{c}-f_{s})}{[V(n_{X}+1)+V(n_{X})]f_{c}f_{s}}$$

The start location of the PN sequence Y is located between the memory address $n_{Y}$ and $n_{Y}+1$, with the offset $\delta_{Y}$. $\delta_{Y}$ can be got by using the above same method. Then, TDOA is written as

(15) $$\tau_{XY}=\tau_{X}-\tau_{Y}=\frac{n_{X}-n_{Y}}{f_{s}/f_{c}}+\delta_{X}-\delta_{Y}$$

Through this snapshot-replay method, even if the receiver cannot stably track the weak signal, TDOAs can be output as long as the short codes of the weak signal can be acquired. However, the accuracy of the TDOA that is calculated by Equation (14) does not take into account the impact of bandwidth, it is not accurate, and there is a large computational error.

### 3.2. Improve the Pseudorange Accuracy Using Taylor Expansion

Taylor expansion is used to improve the computational accuracy of TDOAs. For the sake of analyzing, the factor β is added, and then the receiver RF bandwidth is expressed as

(16) $$B=\beta /T_{c}$$

and Equation (10) is expanded and simplified.

(17) $$R_{L}(\tau )=\frac{1}{2\pi^{2}\beta T_{c}}\left(\begin{array}{l} -2T_{c}\cos(\frac{2\pi \beta \tau }{T_{c}})+T_{c}\cos(\frac{2\pi \beta (T_{c}-\tau )}{T_{c}})+T_{c}\cos(\frac{2\pi \beta (T_{c}+\tau )}{T_{c}})-\\ 4\beta \pi \tau \mathrm{Si}(\frac{2\pi \beta \tau }{T_{c}})+2\beta \pi (T_{c}-\tau )\mathrm{Si}(\frac{2\pi \beta (T_{c}-\tau )}{T_{c}})+2\beta \pi \tau \mathrm{Si}(\frac{2\pi \beta (T_{c}+\tau )}{T_{c}}) \end{array}\right)$$

where $\mathrm{Si}(k)=\int_{0}^{k}\frac{\sin(t)}{t}dt$. However, Equation (17) has Si(k), it is more complicated to calculate. In order to reduce the computational complexity of Equation (17), Taylor expansion is performed on Si(k). Equation (18) is the result of the 24 order Taylor expansion of Si(k). The order of Taylor expansion is also determined according to the output pseudorange accuracy. The larger the Taylor approximation error, then the larger the TDOA calculation error.

(18) $$\begin{array}{l} \mathrm{Si}(x)=x-\frac{x^{3}}{18}+\frac{x^{5}}{600}-\frac{x^{7}}{35280}+\frac{x^{9}}{3265920}-\frac{x^{11}}{439084800}+\frac{x^{13}}{80951270400}-\frac{x^{15}}{19615115520000}\\ +\frac{x^{17}}{6046686277632000}-\frac{x^{19}}{2311256907767808000}+\frac{x^{21}}{1072909785605898240000}\\ -\frac{x^{23}}{594596384994354462720000}+o(x^{25}) \end{array}$$

**Figure 9:**
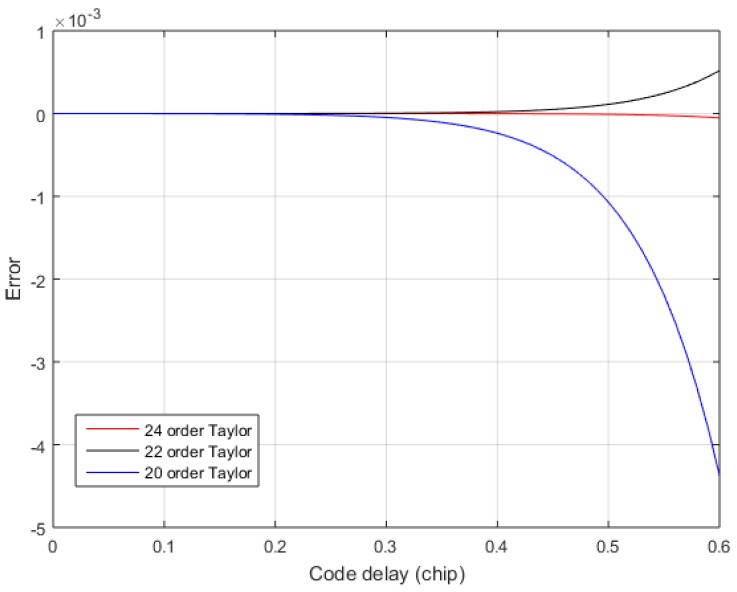
The Taylor approximation error when β=1.6.

**Figure 10:**
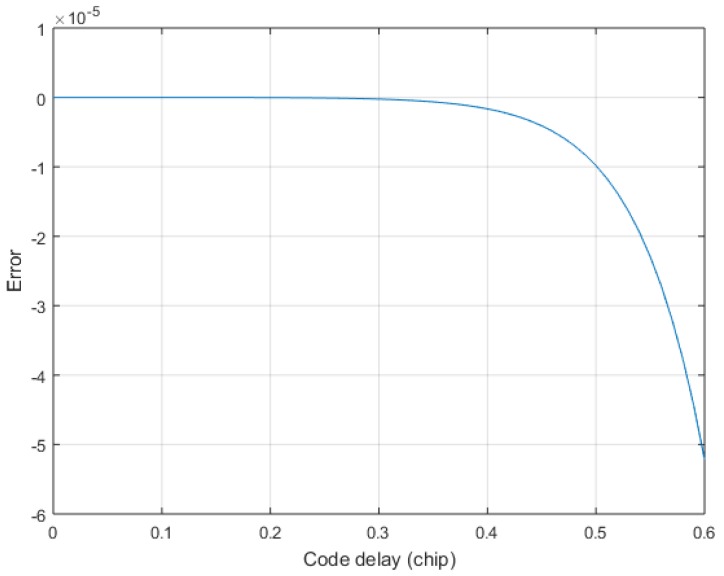
The 24-order Taylor approximation error when β=1.6.

**Figure 11:**
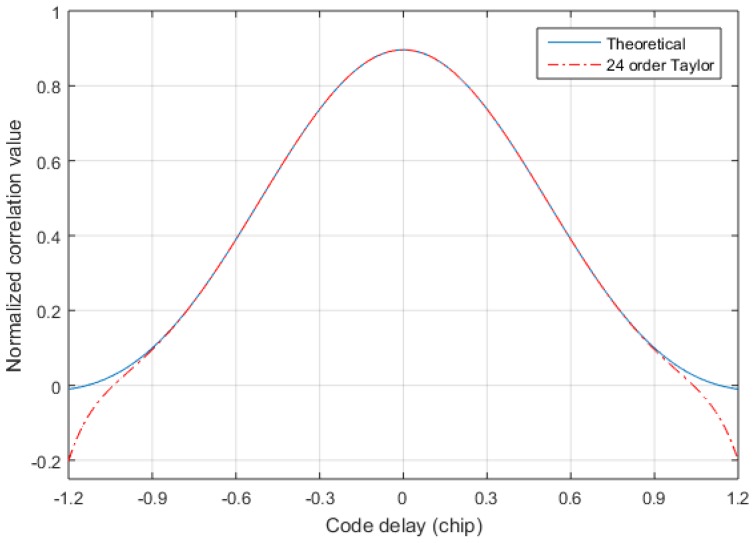
The correlation of 24-order Taylor and the theoretical correlation.

Since the sampling rate $f_{s}$ is at least twice the code rate $f_{c}$, we only care about the fitting accuracy of 0.5 chip. Figure 9 shows the error of the exact solution in the 0.6 chip after the Taylor expansion of Equation (17). In order to observe the 24-order Taylor approximation error clearly, the 24-order Taylor approximation error is plotted separately in Figure 10. From Figure 9 and Figure 10, it is found that the 24-order Taylor expansion satisfies the requirement of the TDOA accuracy, and the error of the 24-order Taylor expansion is less than $1\times 10^{-5}$ within 0.5 chip. Figure 11 shows the correlation of the theoretical calculation and that of the exact fitting calculation of the 24-order Taylor expansion. The fitting function of 24-order Taylor can accurately calculate the correlation under band-limited. Hence, the exact TOA is calculated by using Equations (13) and (17), and then the exact TDOA is retrieved.

## 4. Performance Assessment

Based on the previous discussion, a novel pseudorange measurement algorithm that is based on snapshot is obtained. In this section, simulations and real data tests are performed to verify the feasibility and performance of the proposed algorithm. To achieve a comprehensive assessment of the proposed pseudorange measurement algorithm, Monte Carlo simulations are conducted to compare the proposed algorithm with the regular methods and to verify the reliability of the proposed algorithm. Furthermore, the novel fusion signals are broadcasted by the modified base stations, a base station receiver, and other related equipment is also used to implement the proposed algorithm. Finally, we select several points in a test building to test the positioning accuracy for the static receiver.

### 4.1. Simulations

**Figure 12:**
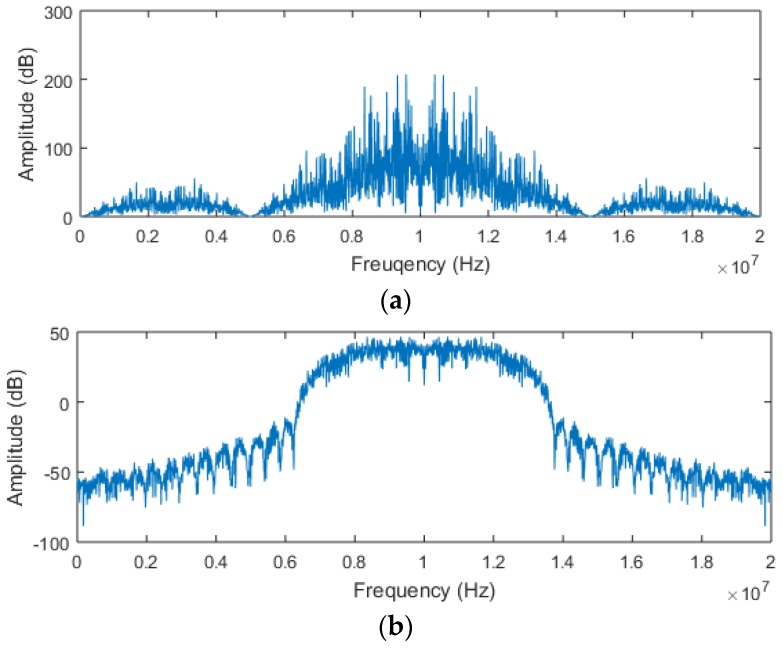
The spectrum of received signals: (**a**) is before filtering and (**b**) is after filtering.

Monte Carlo simulations are utilized for the comprehensive evaluation of the proposed algorithm. All of the simulations are implemented by M-files in MATLAB R2015a. To prove the effectiveness of the novel pseudorange measurement algorithm, comparative tests are performed. The positioning signal of the novel fusion signal adopts Gold codes and is characterized by the parameters in Table 1. For the convenience of analysis, we selected three groups of received signals to test, in which the SNR of a group of received signals is fixed at 0 dB, and the SNR of remaining signals changes from 0 dB to −30 dB. The received signals are simulated by the root-raised cosine filter to compress the infinite bandwidth to within 8 MHz, and the spectrum before and after filtering is shown in Figure 12. 

**Figure 13:**
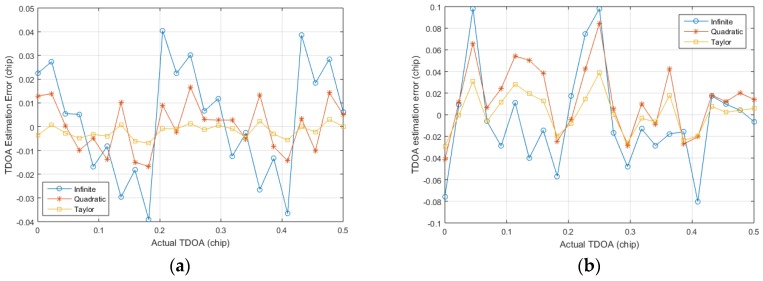
The time difference of arrival (TDOA) estimation errors under different setting code phase differences: (**a**) is SNR = 0 dB and (**b**) is SNR = −15 dB.

The first simulation tests the TDOA estimation error under the same SNR with different code phase deviations. Generating three groups of positioning signals under the limited bandwidth, and then the CMMB signal is superimposed on the generated signals. The code phase differences of the generated signals are known, and finally the Gaussian white noise is added. The stronger positioning signal is acquired and tracked by the regular positioning algorithm, and then the remaining two groups of the positioning signals are stored by the snapshot method described above. The residual carriers of the stored signals are stripped off, and the TDOA of the stored signals are calculated in the following three ways: the infinite bandwidth estimation method described by Equation (14), the quadratic fitting method [14], and the proposed algorithm in this paper. Figure 13 shows the TDOA estimation error under different setting code phase differences of two SNR, and the simulations are repeated 100 times to obtain good statistical properties. It is found that the TDOA estimation error varies with the setting code phase difference, where the fluctuation range of the infinite bandwidth estimation method is the largest. Although the proposed algorithm is affected by SNR, the fluctuation range of the estimated error is the smaller of the other two algorithms. The fluctuation range of the quadratic estimation method is larger than that of the proposed method, and it is greatly affected by SNR. As the short code part is not superimposed with CMMB signals, so this part has high SNR. Assuming that the thermal noise background is −105 dBm, then the 0 dB SNR signal corresponds to the signal strength of −105 dBm, which belongs to the category of weak signal. So the proposed algorithm is still effective for weak signals.

**Figure 14:**
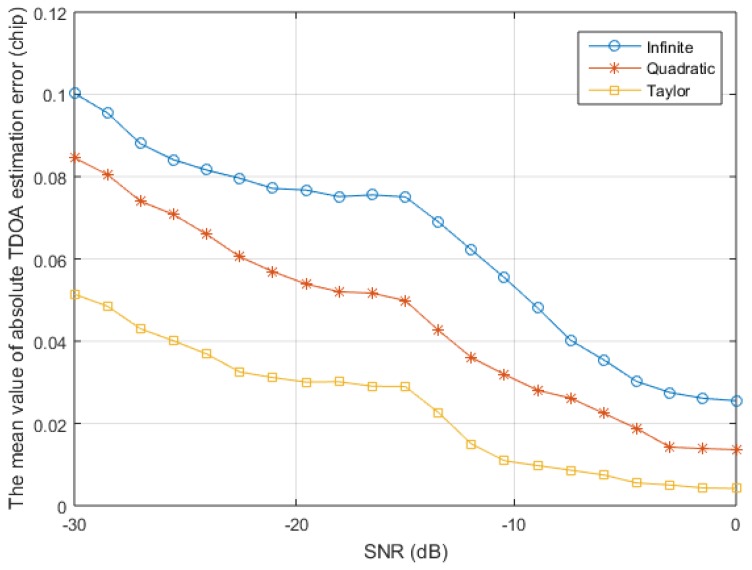
The TDOA estimation errors under different signal-to-noise ratio (SNR).

The second simulation verifies the superiority of the proposed algorithm for various SNR. The signal generation process is the same as the first simulation, and 100 simulations are performed for each SNR with a fixed predetermined code phase difference. The average value of the absolute value of the TDOA estimation error is shown in Figure 14. The three TDOA estimation algorithms that are used for comparison are based on the accurate tracking of one group positioning signal, so the TDOA errors of more than one chip caused by the acquisition error are discarded. It can be seen that the TDOA estimation accuracy of the quadratic estimation method is higher than that of the infinite bandwidth estimation method, and that the TDOA estimation accuracy of the proposed algorithm is significantly higher than these two algorithms under high or low SNR. In addition, the proposed algorithm improves the TDOA estimation accuracy and improves the estimation accuracy of the signal amplitude, which can improve the estimation accuracy under low SNR and finally improve the positioning accuracy of the receiver. 

### 4.2. Real Data Tests

**Figure 15:**
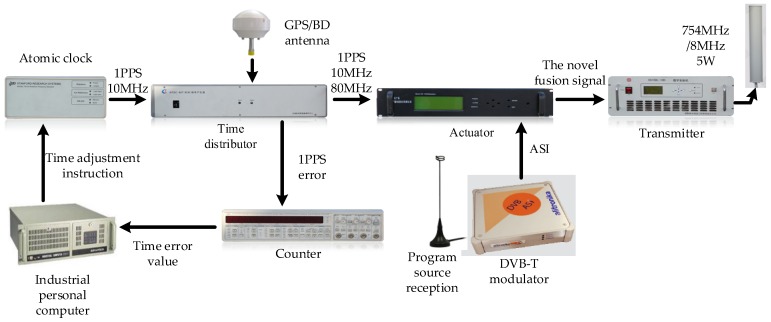
Each equipment of the modified base station.

**Figure 16:**
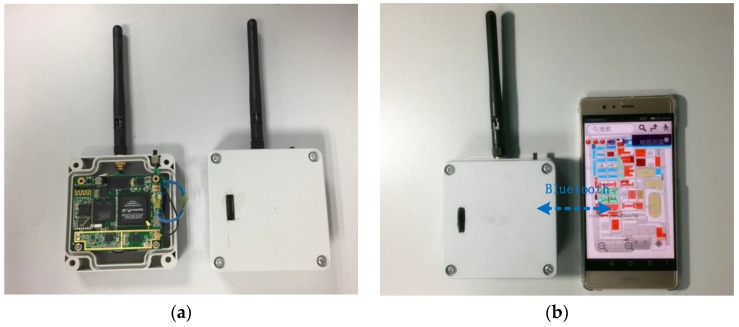
The positioning receiver: (**a**) is the internal and external structure of the receiver, and (**b**) shows that the receiver uploads the related data to the mobile phone through Bluetooth to display.

**Figure 17:**
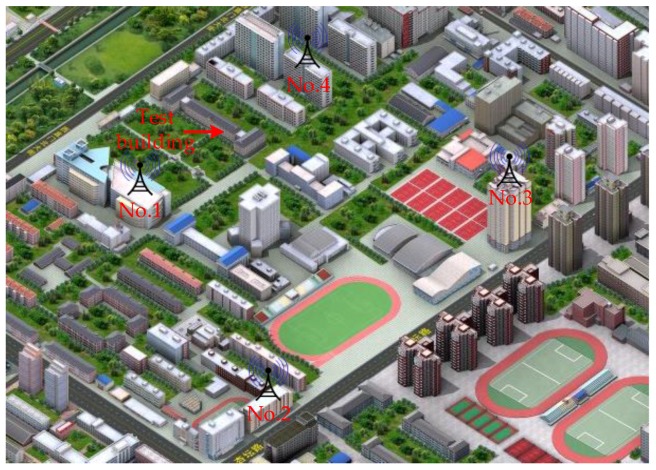
Test environment.

To confirm that the novel algorithm exhibits better actual performance, real data tests are conducted. The comprehensive test platform is shown in Figure 15 and Figure 16. The test platform consists of two parts: the modified base station and the positioning receiver. Each piece of equipment of the modified base station is shown in Figure 15. The output frequency of the atomic clock is 10 MHz, the time distributor receives the GPS/BD signal to ensure synchronization between the modified base stations, and the counter and the industrial personal computer do the corresponding assistance. The synchronization between the modified base stations is up to 5 ns (1 δ). At the same time, the time distributor also generates data messages, including UTC, base station number, air pressure, and base station coordinates. The actuator generates the novel fusion signal, and finally the transmitter transmits the RF signal. Figure 16 shows the positioning receiver that was developed by us. Figure 16a is the internal and external structure of the positioning receiver. The receiver transmits the related data to the mobile phone via Bluetooth and the mobile phone does the map display, as shown in Figure 16b. The positioning receiver utilizes FPGA and ARM architecture for the baseband processing and demodulation, and most of the operations are performed in the FPGA. The intermediate frequency (IF) signal processor can convert the high frequency fusion signal into a zero-digital IF signal with a sampling frequency of 22 MHz. Then, we build an experimental environment on our campus, as shown in Figure 17. Four modified base stations are set up on the roof of four buildings, the 3rd and 4th floor of the other building are selected as the test sites. Finally, the receiver can be used to verify the effectiveness of the proposed algorithm, and to test the positioning accuracy of the entire system.

**Figure 18:**
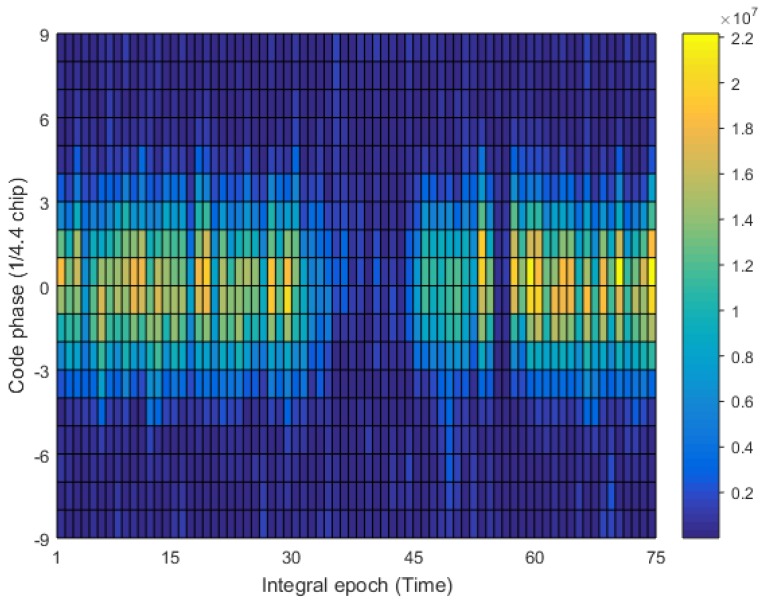
Fading channel distribution.

Firstly, we study the transmission channel of the positioning signal. Only the modified base station No. 2 in Figure 17 is opened, and the receiver collects the RF signal at the walking speed on the 4th floor of the test building. The data collection time is 1.875 s, and analyzes by MATLAB. The fixed residual carrier frequency and the local code rate that is used in the analysis (multiple analysis make these two values close to the real values), and the results are shown in Figure 18. The abscissa denotes the number of integral epoch, and the integral time is 25 ms; the ordinate represents the local replica code phase with the spacing of 1/4.4 chips, and the color temperature represents the non-coherent integral value. It can be found that the correlation peak has a uniform distribution on the time axis, but there is a phenomenon of deep fading and prolonged fading. Therefore, we proposed a pseudorange measurement scheme that is based on snapshot to solve the problem of channel fading. As long as it is possible to store a fragment of received signals that is not impaired by the fading channel, then the accuracy TDOA can be successfully solved.

**Figure 19:**
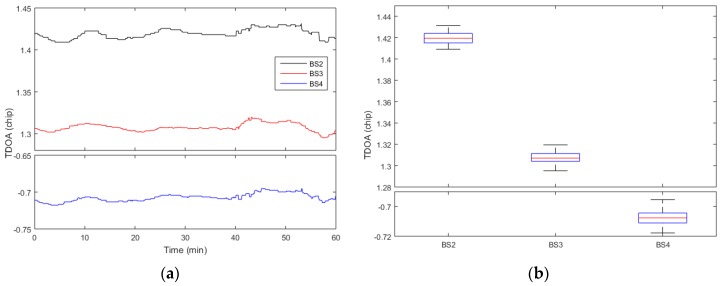
The TDOAs for the No. 1 point of the 3rd floor: (**a**) is the fluctuations with time and (**b**) is the corresponding boxplot.

**Figure 20:**
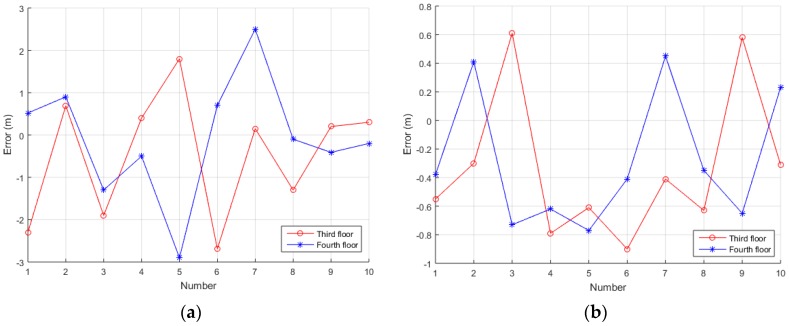
The positioning accuracy of the entire system: (**a**) is horizontal accuracy and (**b**) is vertical accuracy.

Secondly, four modified base stations are opened. We select 10 points to do static positioning test on each test floor in the testing building, the receiver is placed at each test point for one hour. The horizontal positioning uses the proposed algorithm and the corresponding positioning algorithms, and the vertical positioning uses the differential pressure measurement method. Since the system does a custom coordinate system for indoor positioning, the output positioning results are compared with the distance between the selected points and the original point of the corresponding floor. The TDOA method is used for horizontal positioning, and the differential pressure altimetry method is used for vertical positioning. In indoor positioning, in addition to the system error, multipath and not line of sight (NLOS) are the main impact of horizontal positioning results. So, except for the above proposed algorithm, some other adaptive filtering methods are used in the receiver. The calculation of the final horizontal positioning results mainly includes two steps: initial positioning calculation and feature point selection [22]. Using the Chan algorithm, two iterations are performed to get the initial positioning results. Then, the initial positioning error is compensated by the feature points, and the final positioning results are calculated by the Newton iteration method. Figure 19a shows the TDOAs for the No. 1 point of the 3rd floor, and the corresponding boxplot is also shown in Figure 19b. It can be found that the fluctuations of the three TDOAs are within 0.025 chip, and the positioning result fluctuation caused by TDOA measurement is within 1m. The average positioning errors are shown in Figure 20, and the standard deviation of the system positioning results is shown in Table 2 and Table 3. It can be seen that the distance between point and point measured is better than 3 m, and the accuracy of the vertical direction is better than 1 m.

## 5. Conclusions

In this paper, a novel fusion signal is designed to improve the positioning accuracy of the CMMB system, which multiplexes the CMMB and positioning signals in the same frequency band. To reduce the impairments that are caused by complex fading channel and the limited bandwidth, a pseudorange measurement scheme that is based on snapshot is proposed. This algorithm extracts the TDOA estimation from the stored signal sections, and utilizes the Taylor expansion of the autocorrelation function to improve the TDOA estimation accuracy. The algorithm has been tested by numerical simulations and real data. The test results show that the received signals are affected by the fading channel in the actual environment, but the proposed algorithm can significantly reduce the TDOA estimation error for base station positioning receivers when compared with other TDOA estimation algorithms. Finally, the modified CMMB system can achieve meter positioning accuracy.

## Figures and Tables

**Table 1:** Simulation parameters.

Parameter	Value
Slot time, *T_F_*	25 ms
The time of short codes, *T_SC_*	0.136 ms
Bandwidth, *B*	8 MHz
code rate, *f_c_*	5 MHz
Sampling frequency, *f_s_*	22 MHz
Intermediate frequency, *f_IF_*	0 Hz
Residual carrier frequency, *f_d_*_1_, *f_d_*_2_, *f_d_*_3_	1 kHz
Signal length	10 slot times
SNR	0~−30 dB
Data bit transition	Random

**Table 2:** Standard deviation of the 3rd floor positioning results.

No.	1	2	3	4	5	6	7	8	9	10
**RMS(*X*)**	0.76	0.64	2.92	0.75	0.05	0.65	0.23	0.07	0.15	1.25
**RMS(*Y*)**	0.74	1.07	0.72	0.71	0.04	0.39	0.16	0.11	0.48	0.68
**RMS(*Z*)**	0.48	0.63	0.31	0.44	0.52	0.43	0.61	0.51	0.62	0.51

**Table 3:** Standard deviation of the 4th floor positioning results.

No.	1	2	3	4	5	6	7	8	9	10
**RMS(*X*)**	0.29	0.73	0.35	0.94	0.67	1.01	0.69	0.87	0.29	0.46
**RMS(*Y*)**	0.17	0.94	0.16	1.08	0.42	0.55	0.41	0.97	0.84	0.65
**RMS(*Z*)**	0.51	0.47	0.34	0.39	0.39	0.47	0.57	0.36	0.59	0.57
